# From Dyadic Distance to Space in Family Networks: Reciprocity of Family Support in Switzerland

**DOI:** 10.1111/1468-4446.70025

**Published:** 2025-08-18

**Authors:** Gil Viry, Andreas Herz

**Affiliations:** ^1^ School of Social and Political Science University of Edinburgh Edinburgh UK; ^2^ Deutsches Jugendinstitut München Germany

**Keywords:** distance, family relationships, networks, reciprocity, space, support

## Abstract

Due to their geographical dispersion, many families face challenges in exchanging support over long distances. While family theories emphasise the importance of a systemic approach to family relationships, reciprocity—a core feature of these relationships—is still predominantly studied within specific dyads, such as the parent‐child relationship, rather than within the broader family network and its spatial context. This study addresses this gap by examining whether family members reciprocally exchange material and emotional support, and how these exchanges relate to spatial characteristics at three levels: the individual (past migration, degree of urbanisation), the dyadic tie (physical distance between members) and the network (spatial dispersion). Using a national sample of 549 adults living in Switzerland, who named important family members and identified available support, we apply a multilevel network approach. Results show that only reciprocity in material support declines with residential distance when controlling for both in‐person and remote contact. Moreover, reciprocity is more likely in large, tightly‐knit families, and—specifically for emotional support—in spatially dispersed ones. This last finding suggests that reciprocating emotional support is a key mechanism through which families maintain long‐distance relationships. Another takeaway is that cultivating mutually supportive ties must be understood not only through dyadic distance and contact between individual members, but in relation to the spatial and network context of the family as a whole.

## 1 Introduction

Reciprocity in support exchange has long been viewed as a key mechanism of social integration and solidarity that binds social groups together through a complex web of interdependencies (Adloff and Mau 2006). Reciprocity in social relationships may either be direct (giving back to the individual who provided help) or indirect (giving to a third person through group norms). Social theories of reciprocity emphasise the decisive role of *social contexts* in facilitating or inhibiting participation in reciprocal exchanges (Hollstein 2005; Offer 2012). Individuals embedded in cohesive and supportive groups are likely to reciprocate more than in less‐cohesive ones because of cost‐benefit calculation, moral obligation, better communication and coordination, but also because giving, receiving and reciprocating are ways of creating, maintaining and regenerating social bonds within groups. In this sense, reciprocity is both a result of and a contributor to group cohesion.

In the context of geographical dispersion of families, a key issue is whether space, particularly physical distance, may hinder support provision and reciprocity (Halatcheva‐Trapp et al. 2019; Hallman 2010; Small and Adler 2019). Despite the widespread use of mobile technologies, evidence shows that the further away family and friends live, the less they communicate, in person but also remotely, and socialise together, with evidence of rapid nonlinear decay as distance increases (Axhausen and Frei 2007; Mok et al. 2010). But we also know from transnational family studies that migrants and their families can exchange affection and care, and sustain a sense of being emotionally close, with others living far away (e.g., Baldassar 2007; Baldassar and Merla 2013). In this study, we adopt the perspective that cultivating family bonds over distance involves more than simply overcoming physical separation through person‐to‐person communication technologies and visits with individual family members (Bissell 2013; Holdsworth 2013). It also requires navigating spatial dispersion at the family level, for example by coordinating family gatherings, fostering a sense of togetherness, and sustaining family solidarity across places through shared routines, normative expectations and collective memories. Such family dynamics are well illustrated in the experiences of diaspora, military and seafaring families (Heiselberg 2017). From this perspective, we argue that it is essential to consider the spatial dispersion of the family as a whole, rather than focusing solely on dyadic distances between individual members. Accordingly, we take into account spatial characteristics at the individual, tie and network levels when analysing how space shapes and is shaped by reciprocity in family relationships. While these characteristics influence and help explain the quality of long‐distance family relationships, reciprocity can also sustain family relationships over distance.

Yet, only sporadic research has been conducted on reciprocity within family networks, usually in a cross‐cultural approach (Agree et al. 2005; Komter and Schans 2008). Many studies have focused on specific family dyads, namely parents and children. Space is therefore often limited to dyadic distance (or spatial proximity), overlooking possible structural effects at the family level (van Gaalen et al. 2008; Widmer 2016). In quantitative studies, physical distance is often treated as an external factor of social relationships, rather than as an inherent characteristic that actively shapes and defines individuals (e.g., past migration), relationships (e.g., cross‐border ties) and families (e.g., transnational families). Consequently, many quantitative studies, although with some important exceptions (see Lubbers et al. 2020 for a review; Madhavan et al. 2017), examine the negative influence of distance on specific relationships considered in isolation rather than as embedded in the family as a unit (see e.g., Schafer and Sun 2022).

The present study addresses this gap by investigating whether reciprocal exchange of material and emotional support in family ties varies with space considered at three levels: individuals (past migration, degree of urbanisation), ties (physical distance) and networks (family spatial distribution). We use a national sample of Swiss adults who named important family members and identified available support (who would you help and who would help you in hypothetical situations). Adopting a multilevel modelling approach with personal (egocentric) network data, we expand the study of reciprocal exchanges in family dyads to family structures. With this network approach, we consider the family as a set of personal relationships (i.e., a personal network) that is spatially embedded and not reducible to the properties of separate relationships (Widmer 1999). We conceptualise space as endogenous characteristics of individuals, ties and networks. When family members are geographically dispersed, communicating needs, coordinating support and maintaining solidarity norms entail considerable effort that may vary substantially across individuals, relationships and families, depending on their particular histories, resources and cultural expectations. This, in turn, may affect who is considered being part of one's family (Mason 2004). While some families may cultivate solidarity across broader spatial scales through visits and telecommunication, others may be more rooted in local routines—contexts in which sustaining relationships and providing support provision over distance may be less likely.

We use data from Switzerland. This country is characterised by weak national family policies, with family issues primarily viewed as private matters. Grandparents play an important role in informal childcare. The local organisation of schools, childcare, health and welfare within a federal structure strengthens place attachment and deters people, especially parents, from moving between regions. The well‐developed transport system reinforces this trend by facilitating interregional travel rather than relocation. At the same time, Switzerland has been characterised by an important international immigration since World War II, especially in metropolitan areas, fuelled by strong economic growth. A third of the Swiss resident population has one or both immigrant parents and a quarter was born abroad, more than any other European countries (Piguet 2013). These features are likely to offer important variation in the ways individuals and families negotiate long‐distance relationships.

The article continues by reviewing literature on reciprocity within families, and then on the role of space on family relationships and family support. After describing the data and methods, we present descriptive results followed by the results of the multilevel analysis. The final section discusses the theoretical implications of the findings and the importance of studying social relationships in spatial and network contexts.

## 2 Reciprocity in Families

We distinguish three main theoretical approaches to reciprocity: the utilitarian approach, the normative approach, and the relational approach rooted in the work of Marcel Mauss. In the first one, self‐interested individuals are more likely to help people who are expected to reciprocate favours and help them meet their own needs in the short or longer term (Blau 1964; Offer 2012). Failing to reciprocate induces dependency and may lead to exclusion from future exchanges. Supportive groups reduce the uncertainty of not receiving something back, making individuals more likely to engage in reciprocal exchanges out of rational self‐interest. In the normative approach, people are more likely to reciprocate where there is a moral obligation to do so (Gouldner 1960; Hollstein 2005; Komter and Schans 2008). Like in the utilitarian perspective, cohesive groups foster reciprocation. Good communication about needs and a strong sense of belonging and solidarity can enforce social obligations and trust, which morally bind people together and contribute to mutual support. In the relational approach developed in the tradition of *The Gift* (Mauss 2001 [1925]), reciprocity is not merely a consequence of self‐interest or moral duty, but a means of creating, maintaining, and regenerating social bonds (Adloff and Mau 2006). In cohesive groups, reciprocal exchanges are grounded in embedded and ongoing social obligations, where the acts of giving, receiving and returning favours are essential to sustaining and reaffirming group cohesion, trust and mutual belonging, beyond the individual interests or moral considerations of the actors. From this perspective, the relationship itself is more central than the transfer.

Hollstein (2005) notes that family relationships do not necessarily rest on patterns of reciprocity to exist. Due to family norms and roles, the bond to a sister or son‐in‐law, for example, can be considered of significance by individuals, while being asymmetrical or even unsupportive. The unequal distribution of resources between parents and dependent children also conflicts with the norm to reciprocate. Parents support their dependent children by the law and often continue long after the children left the household. Most research on reciprocity in family relationships has however focused on the parent‐child bond by examining whether adult children support their elderly parents as repayments for the support received earlier in life (e.g., Leopold and Raab 2011). Less is known about reciprocity within the broader set of family relationships. This aspect has been discussed through the concept of generalised reciprocity: the idea that reciprocity does not require immediate return, return in the same domain or return to the same person. Although this concept has long been discussed in family research because of the strong normative framework of mutual support attached to families, empirical work is relatively limited (Hollstein 2005; Molm et al. 2007). Generalised reciprocity aligns closely with Mauss's relational perspective, where maintaining social relationships takes precedence over the strict balancing of exchanges between individuals. Support is given without an explicit expectation of immediate or equivalent return. The act of returning may be so deferred in time that the orientation is less toward exchange equity than toward actors' situational needs and resources. Equity is considered in the long term and mutual trust exists that help will be returned when the opportunity arises, for example in case of hardship. This however does not imply that the act of giving, receiving and reciprocating is disinterested. Evidence from family studies suggests that people evaluate their own and others' needs and resources when asking for and providing assistance (Hansen 2005; Nelson 2000). In situations in which individuals have few resources to share, like in low‐income families, reciprocity can be a burden and a source of withdrawal from family networks (Offer 2012). Conversely, relations of mutual indebtedness and dependency are established with those who help in return, leading to relationships of trust and obligation. It is precisely the presence or absence of this characteristic of family ties that we examine in this study.

Plickert et al. (2007) is one of the rare studies considering reciprocity within personal networks of family and friends. Using data from 29 respondents surveyed in 1968 from a borough of Toronto in Canada, the authors examined whether the provision of emotional support, minor and major services from respondent to network members is associated with the provision of the same or different types of support in the other direction. Analysis showed that people tend to get the same kind of support that they give (e.g., minor services for minor services). Tie characteristics, such as frequency of contact (either in‐person or by phone), role relationships (e.g., friend, parent) and tie strength (social closeness) had relatively little effect on reciprocity, while these factors did largely affect the *one‐way* provision of support. The effect of residential proximity was also tested. Respondents often reciprocated minor services with people living in the same neighbourhood. Neighbouring had a stronger effect than residential distance. Considering network effects, only the network size had a small effect, with a higher probability of reciprocating emotional support in large networks. The authors concluded that reciprocity has ‘become less rooted in the social control and rewards of the group and become more a product of trust within ties’ (Plickert et al. 2007, 406). Reciprocity would strongly depend on the initial giving of support that is strongly influenced by tie characteristics, such as residential proximity. Departing from this view, we argue in this study that mutually supportive relationships are shaped not only by the characteristics of individual ties, but also by the broader configuration of the family network, including its spatial dispersion. Focusing solely on dyadic distances between family members overlooks the spatial pattern of the entire family and the ways in which support exchanges are embedded within the family's spatial organisation (Holdsworth 2013). For example, the relationship between two siblings living nearby may carry different forms of support and expectations depending on the spatial distribution of the wider family. When other relatives are geographically close, responsibilities and support are distributed across many family members. In situations where the rest of the family lives far away, the local siblings may become central to maintaining family cohesion locally. Their relative proximity can then strengthen their bond, but also place more strain on it. In this sense, the spatial dispersion of the family as a whole provides important contextual information for understanding the dynamics of family solidarity and reciprocity. Our approach thus extends beyond pairwise relationships to capture the wider spatial structure in which these ties are situated.

## 3 Family Relationships and Support in Space: Research Questions

A growing literature highlights the importance of considering space and spatial mobility as central features of contemporary families (Halatcheva‐Trapp et al. 2019; Hallman 2010; Holdsworth 2013). Spatial contexts shape opportunities for social interaction through various mechanisms, with physical proximity being the most widely studied (Small and Adler 2019). This literature recognises that space is not a ‘container’ in which family activities happen and which affects family life ‘from the outside’, as when considering that physical distance undermines family cohesion and solidarity. Instead, space is conceived as inherently related to, and constitutive of, family life and family identity. It actively shapes and defines actors, social relationships and networks through spatially embedded experiences, histories, identities, understandings, etc., which may be lived individually or shared collectively. In this study, we examine the role of space in reciprocity at three levels within a single analysis: individuals (past migration, degree of urbanisation), ties (dyadic distance) and networks (spatial dispersion of the family).

At the individual level, past mobility matters because it differentiates between those who have moved (possibly together with other family members) and those who remained rooted (while others possibly moved away from them). In this study, past migration is defined as having experienced long‐distance residential mobility, either within Switzerland or from abroad. While care and support can be sustained over long distances through family visits and telecommunications (Baldassar 2007; Baldassar and Merla 2013), skills and resources prove to be crucial for migrants to overcome the negative consequences of physical separation. The ability to support family members may be lower among migrants with limited resources (e.g., little money to travel). Support exchange may also depend on the reasons and circumstances of the move (Hagge and Schacht 2024). Migrants who moved away to distance themselves from negative family relationships may therefore be less willing to support family members than non‐migrants. Conversely, for a given residential distance, migrants may be more willing than non‐migrants to exchange support with specific family members. Migrants more often actively chose to move while maintaining ties, whereas non‐migrants may feel more passive in the separation—having stayed rooted while others moved away—which could affect their willingness or perceived obligation to provide support.

Residential context may also influence reciprocity in support exchanges. Urban residents typically benefit from greater mobility resources, including better transport infrastructure (Urry 2007), which may facilitate support exchanges with geographically dispersed family members. Meanwhile, US studies suggest that rural families are more likely to rely exclusively on kin for support than urban families, reflecting differences in social norms and institutional service availability (Beggs et al. 1996; Hofferth and Iceland 1998). However, in small, wealthy and well‐connected countries like Switzerland, rural‐urban disparities in support access may be attenuated by access to personal cars. Additionally, digital technologies could mitigate distance barriers for intangible support (e.g., emotional or financial assistance) across all residential contexts. Taken together, we expect little differences in support reciprocity between urban and rural settings.

This leads us to the first research question: *How do past migration and degree of urbanisation influence reciprocity in* *support exchanges between individuals and their family members?*

Studies that examined the spatial distribution of core personal networks have shown that the further away the respondents and their network members live, the less they communicate—in‐person but also remotely—and socialise together, with evidence of rapid (nonlinear) decay as distance increases (Axhausen and Frei 2007; Mok et al. 2010). Support provision is less sensitive to distance than contact, but a similar trend is observed for some forms of tangible support (e.g., Mok and Wellman 2007). However, this perspective tends to treat space primarily as a challenge—an external factor that must be overcome—rather than as an inherent characteristic of individuals. Yet, the evidence for such decay is not unequivocal. Some studies on personal networks found that long‐distance ties can be particularly strong relationships due to a selection effect: only the strongest ties survive greater distances (Cachia and Maya‐Jariego 2021; Viry 2012). Less tangible forms of support, such as emotional and financial aid, proved to be exchanged with loved ones living far away (Baldassar and Merla 2013; Mok and Wellman 2007). Research also suggests that relationships with extended kin, neighbours, co‐workers and casual friends are more dependent on physical proximity than relationships with parents, children and siblings—close friends often lying in between (e.g., Lubbers et al. 2010; Mulder and van der Meer 2009). These differences are usually explained by tie strength but also normative obligations and the density of connections that enforce mutual obligations, although the latter explanation is rarely tested empirically. This literature not only emphasises the continuing importance of places and spatial proximity in the age of mobile technologies, but also acknowledges that individuals consider as important some relatives with whom they interact little due to distance constraints.

Our second research question is: *How does dyadic distance influence reciprocity in* *support exchanges between family members?*

In this study, we argue that mutual support in family relationships is shaped not only by the physical distance between two family members, but also by the structure and spatial dispersion of the entire family network. While dyadic distance captures how far apart two relatives live, network‐level dispersion reflects the broader spatial configuration of the family, which can shape support expectations, the availability of alternative helpers and the burden placed on specific relationships. Whether driven by instrumental motives, moral obligations, or embedded in ongoing social obligations that strengthen group cohesion, reciprocity is expected to be more likely to occur in family relationships when these are embedded in cohesive family contexts. While geographical propinquity of family members may facilitate these conditions, evidence suggests that spatially dispersed families who sustain a family identity and sense of belonging across geographical distances may also represent a favourable context for the provision of mutual support (Baldassar and Merla 2013; Lubbers et al. 2020). In Mauss's relational perspective, reciprocating support may serve as a way of sustaining family relationships and actively ‘doing family’ over distance—a dynamic that may become even more important when family members are physically separated.

Our third research question is: *How does the spatial dispersion of family networks influence reciprocity in* *support exchanges between family members?*

## 4 Data and Methods

### 4.1 Sample

We use data from the MOSAiCH‐ISSP 2013 survey that consists of a nationally representative sample of 1237 adults living in Switzerland who completed a questionnaire administered by in‐person interviewers (CAPI) (response rate: 51.4%) (for more information, visit https://forscenter.ch/projects/mosaich/). Among them, 754 persons (61%) participated in a follow‐up survey by returning a self‐administered questionnaire by post that included a family network module. Of those, 648 respondents (86%) cited at least one person in their family network and answered questions about social support. The 549 persons (73%) who had valid data on all study measures constitute the analytic sample. We tested sampling bias by comparing these observations and those of the ISSP original sample (*n* = 1,153, see Appendix Table A1). The results indicate only few statistically significant differences—most of them being commonly observed bias due to attrition in follow‐up surveys. Women, people older than 50 years, with a university degree, from the upper‐grade service class, Swiss citizens and those living in French‐speaking Switzerland were more likely to participate in the follow‐up survey and answer the questions on family support. There is no significant effect of the partnership status or the number of children.

### 4.2 Egocentric Network Data and Multilevel Approach

Information on family relationships and support was collected applying an egocentric network approach. The interpersonal environment of individual respondents is seen from their point of view: ‘[a]n ego‐centred network consists of a focal actor, termed ego, a set of alters who have ties to ego, and measurements of the ties among these alters’ (Wasserman and Faust 1994, 42).

We apply a network design where respondents provide information about their direct (ego‐alter) and indirect (alter‐alter) relationships following so called cognitive social structures (CSS) (Widmer 1999). Respondents were first prompted to give a list of people they currently consider important members of their family. ‘Who are the *people you currently consider as important family members*? By important family members, we mean the people who have played a *role*, either *positive* (these people helped or supported you) or *negative* (they annoyed you), over the past 12 months, and who *you consider being part of your family*.’ In defining their own family, participants could include individuals who were not related to them by blood or marriage, such as a friend or live‐in nanny. They could name up to 11 family members (alters). They were then asked to provide basic information about these alters (e.g., age, sex) and about the relationships they had with them, such as frequency of contact and role (child, partner, etc.).

Respondents were further asked about access to support in all relationships, that is in both ego‐alter and alter‐alter relationships. Measuring access to support—who would help you in a hypothetical situation—is often preferred over actual use of support (who has helped), which conflates need and access. Social support can generally be divided into several dimensions, such as material, emotional and social companionship. Material support is defined as supplying material or tangible help through goods (including money) or minor or major services. Emotional support comprises giving advice and talking about personal problems, whereas social companionship means the sharing of social activities (Van der Poel 1993). In MOSAiCH, only questions about access to material and emotional support were included, in which material support captures both financial and practical help. For material support, the following items were used: (1) ‘*Among the family members you listed, who do you think would provide you material* *support or give you a helping hand when you are facing difficulties, such as financial shortage, childcare, shopping, transport, housework?’* (alter‐to‐ego support); (2) ‘*Among the family members you listed (including yourself), who do you think, would provide material* *support to >> FIRST PERSON NAMED << or give her/him a helping hand when she/he is facing difficulties, such as financial shortage, childcare, shopping, transport, housework etc.?’* (ego‐to‐alter and alter‐to‐alter support) and so on for all the alters named. For emotional support, the following items were used: (1) ‘*Among the family members you listed, who do you think would give you emotional* *support when you experience some everyday problems (for example when you are sad or had a hard day, who can* *support, comfort or calm you)?’* (alter‐to‐ego support); (2) ‘*And among your family members (you included), who do you think would give emotional* *support to >> FIRST PERSON NAMED << when they experience some everyday problems (for example when they are sad or had a hard day, who can* *support, comfort or calm them)?’* (ego‐to‐alter and alter‐alter support) and so on for all the alters named. Respondents were additionally asked about all ego‐alter and alter‐alter ties whether they think ‘person X often gets on person Y's nerves’ (annoying) and whether they think ‘person X could make person Y change his/her mind’ (influence).

We apply a multilevel regression analysis using ego‐alter support exchanges as the units of analysis. Although it is conceivable to use the full set of ego‐alter and alter‐alter ties among family members in specific models for social networks (see e.g., Vega Yon et al. 2021 on a method for modelling pooled small networks), we chose a multilevel egocentric approach for both conceptual and methodological reasons. Ego‐alter and alter‐alter ties are not strictly equivalent, as egocentric data are centred on the respondent, which introduces a reporting bias and limits the availability of alter‐level and dyadic information across all possible ties. The multilevel framework we employed allows for the simultaneous analysis of predictors at the ego, tie, and network levels—an important advantage for capturing the layered structure of personal networks and for investigating how individual characteristics shape reciprocity in family ties. Alter‐alter support exchanges and residential distances were respectively used to construct network density and network spatial dispersion as network‐level predictors.

Networks are specified as a two‐level model consisting of ego‐alter family ties (Level 1) nested within egocentric networks (Level 2). Both alter's attributes (e.g., alter's gender) and tie attributes (e.g., tie duration) are analytically treated as Level 1 variables. Both ego's attributes (e.g., gender, past migration) and network attributes (e.g., density, spatial dispersion) are analytically treated as context for ego‐alter ties (Level 2 variables). We examine separately reciprocity in material and emotional support (outcome variables), because exchanges of material services (e.g., physical care) often require physical presence (and therefore spatial proximity or travel), compared with affection and emotional support that can also be exchanged through telecommunications. While hierarchical models are widely used for nested data structures and were pioneered for egocentric networks in the late 1990s (Snijders et al. 1995; van Duijn et al. 1999; Wellman and Frank 2001), its application to family studies is still limited (Viry and Herz 2021).

Multilevel modelling (MLM) is well‐suited to account for the nesting of family ties within family networks, while also examining how tie reciprocity varies with spatial characteristics at the individual, tie and network levels. MLM adjusts standard errors for the clustering of alters and ties in egocentric networks and assesses the network effects, net of the effects of single dyads. Reciprocity in material and emotional support is measured as direct (one‐to‐one) mutual exchange of support in ego‐alter dyads. While we do not examine support exchange through more complex pathways involving more than two actors (e.g., giving to our children what we receive from our parents), the analysis accounts for the fact that tie reciprocity varies with the size, structure and spatial dispersion of the family network.

### 4.3 Ego‐Level and Network‐Level Measures

Table 1 presents the socio‐demographic characteristics of the *n*_*e*_ = 549 respondents (egos) of the analytic sample. The mean age is 48.34 years and 53% of respondents were female. Respondents were asked to report the municipality in Switzerland where they currently live and the one where they lived at age 14 or the country if they lived abroad. Switzerland has more than 2000 municipalities and the area covered by each is usually small (median: 7.3 square kilometres). We used routing software to calculate road distances between the centres of municipalities in order to generate a measure of past migration. Respondents living more than 40 km from where they resided at age 14, as well as those who were born abroad, were classified as migrants, representing 36% of the sample. While any distance threshold involves a degree of arbitrariness and is context‐dependent, we selected 40 km, as it roughly corresponds to the size of a large labour market area in Switzerland based on commuting flows (OFS 2019). This distance allows for occasional visits but typically not for daily interaction. Although we do not have information on whether the alters themselves moved, this measure enables us to compare support exchanges between respondents (egos) who have migrated and those who have remained in their place of origin. Degree of urbanisation of the residential municipality derived from Eurostat classification in three categories: (1) cities (densely populated areas); (2) towns and suburbs (intermediate density area) and (3) rural and mountainous areas (thinly populated areas) (for more information visit http://ec.europa.eu/eurostat/ramon/miscellaneous/index.cfm?TargetUrl=DSP_DEGURBA). Social class was measured based on the last occupation using Oesch's class scheme in five categories (Oesch 2006). We excluded the level of education from the final analysis since education is strongly correlated with social class and has little effect on reciprocity. Health status was measured with the question: ‘In general, would you say your health is (1) poor, (2) fair, (3) good, (4) very good and (5) excellent’ with a mean value of 3.53. We excluded respondents' personal and household income from the analysis, because their effects were statistically insignificant and because including them would markedly decrease the sample size.

**TABLE 1:** Respondent (ego) and network characteristics (Level 2) (*n*_*e*_ = 549).

Variable	Categories	Min	Max	%	Mean	SD
Respondent characteristics						
Gender						
	Female			53		
	Male			47		
Age		18	89		48,34	16,45
	18–30 years			18		
	31–50 years			36		
	51–65 years			29		
	66–100 years			17		
Health status (1: Poor, 5: Excellent)		1	5		3.53	0.90
Social class						
	Unskilled workers			9		
	Skilled workers			30		
	Small business owners			11		
	Lower‐grade service class			25		
	Higher‐grade service class			25		
Past migration						
	Distance < 40 km			64		
	Distance 40+ km			36		
Degree of urbanisation						
	Rural areas			27		
	Towns and suburbs			46		
	Cities			27		
Network characteristics						
Size (number of family members including ego)		3	12		7.51	2.77
	Small	3	6	40		
	Medium	7	9	34		
	Big	10	12	26		
Density (ratio of existing to possible ties)		0	1		0.41	0.21
	Low	0	0.33	38		
	Medium	0.33	0.5	32		
	High	0.5	1	30		
Spatial dispersion (average distance between all family members)						
	All family members live in the same household			13		
	< 5 km			13		
	5–20 km			20		
	20–100 km			26		
	100+ km			28		

Table 1 also presents the descriptives of the network size, density and spatial dispersion. Network size is the sum of all family members including the respondent. The mean network size is 7.51 (SD = 2.77; min = 3; max = 12). A minimum network size of 3 was necessary for calculating a network density score, so we excluded 29 respondents (about 4% of the sample) who cited only one family member. Network density is measured as the ratio of existing ties to all possible ties in a network and ranges between zero (no existing ties) and one (all possible ties exist). We calculated the density score based on alter‐alter ties only, excluding ego‐alter ties, since density was used as predictor of reciprocity in ego‐alter ties. We used the four kinds of relationships available in the data: material support, emotional support, influence and annoying, to capture the degree of connectivity within the family according to all these dimensions. For this, we assigned tie values ranging from zero to 4 with zero indicating that no kind of relationship was existing and 4 indicating that all kinds of relationships were existing (in the respondent's perspective). Network density was then calculated by dividing the sum of alter‐alter tie values by its maximum possible value (i.e., the number of alter‐alter ties multiplied by 4). The mean density is 0.40 (SD = 0.21; min = 0; max = 1). A density measure based solely on support ties, excluding influence and annoying, was also tested and yielded similar results. The network spatial dispersion is measured by the mean distance in kilometres between the residences of all network members (both ego and alters). For each alter, respondents were asked to indicate whether this person resided in the same household and, if not, in what municipality if the person lived in Switzerland or in what country if they lived abroad. We imputed the residential distance between any two network members using a routing software calculating the road distance between municipality centres. An important data limitation is that the exact location of alters living abroad is unknown (*n*_*a*_ = 385 alters, 12% of ties, which concerns *n*_*e*_ = 136, about 25% of egos). We set the distance to 500 km between people living in different countries, which is the largest possible distance within Switzerland. This strategy overestimates the distance for alters living close to the Swiss border in neighbouring countries (France, Germany, Italy and Austria) and underestimates the distance for alters living far away. This issue is partly mitigated by using broad categories with large dispersions (e.g. 100+ km). Distances between alters living in the same country outside Switzerland were set to zero, assuming that many of these people lived in the same place (e.g., mother and father living abroad). The spatial dispersion was then calculated by averaging the ego‐alter and alter‐alter distances in each network, and by grouping networks into 5 categories: (1) all family members live in the same household; (2) mean distance less than 5 km; (3) 5–20 km; (4) 20–100 km and (5) 100+ km. We tested three alternative measures of spatial dispersion and all of which yielded similar results, including median distance and a classification based on the residential concentration or dispersion of alters across areas.

### 4.4 Tie‐Level and Alter‐Level Measures

Reciprocity of family relationships in material and emotional support are the two outcome variables of interest. They are measured as the presence of a two‐way exchange (symmetrical tie) and coded as 1 when the ego reports that he/she gives one kind of support and gets back the same kind of support from the alter. All other situations are coded as 0, that is only ego provides support, only alter or neither of them (non‐supporting ties). We decided to keep non‐supportive ties (3.6% of the total number of ego‐alter ties) in the calculation of reciprocity because they are considered to be important family relationships by the participants. In this conceptualisation, reciprocity is understood as a central feature of strong and enduring social relationships—one that may or may not characterise a given family tie at a particular point in time. We therefore examine whether ties between respondents and their family members are reciprocal (as opposed to non‐reciprocal or non‐supportive), considering that support may be available because of past actions (e.g., indebtedness), interest (expectations of future rewards), moral obligation, or as a means of maintaining the bond.

While relationships are considered reciprocal when support flows in both directions, our data do not capture the amount, value, or perceived fairness of the exchanges. Some participants may view relationships as reciprocal, yet still perceive them as asymmetrical, feeling they receive or give support but not enough relative to their needs or their actual or potential contribution.

Because reciprocity can occur with the exchange of one kind of social support for the other, sensitivity analysis also tested a broader definition of reciprocity in material *or* emotional support, that is ego would give one or both forms of support to and would receive one or both forms of support from the alter (see Appendix Table A2). For comparison, we also tested models that predict whether a tie is non‐reciprocal in the sense that ego would give one or both forms of support to and would not receive any support from the alter (see Appendix Table A2). The opposite situation where ego would receive one or both forms of support and would not give any support to the alter was rare and the multilevel models did not converge. Because results from these sensitivity analyses did not substantially differ from the main analysis, we only report them when useful.

The *n*_*e*_ = 549 respondents reported a total of *n*_*a*_ = 3345 ego‐alter relationships. Table 2 shows the percentage of reciprocal relationships for material and emotional support among those relationships. On average, participants reported that they would reciprocally exchange material support with 73% of cited family members. This means that almost three quarters of family relationships would mutually exchange help with financial shortage, childcare, shopping, transport, housework, etc. and more than a quarter would not. In terms of emotional support, about two thirds (65%) of the family ties are reciprocal. The rate of reciprocity raises to 82% for relationships involving any exchange of social support, whether in the form of emotional or material support.

**TABLE 2:** Characteristics of alters and ego‐alter relationships (Level 1) (*n*_*a*_ = 3345).

Variable	Categories	Min	Max	%	Mean	SD
Reciprocity in material support		0	1		0.73	0.44
Reciprocity in emotional support		0	1		0.65	0.48
Alter gender						
	Female			54		
	Male		46			
Alter age		1	98		43.96	22.46
	0–17 years			14		
	18–30 years			17		
	31–50 years			28		
	51–65 years			22		
	66–100 years			19		
Ego‐alter residential distance						
	Same household			27		
	Same municipality			16		
	Another municipality < 20 km			21		
	Another municipality > 20 km			24		
	Other country			12		
Alter's role relationship with ego						
	Child			21		
	Partner			13		
	Father/mother			14		
	Brother/sister			15		
	Extended family			18		
	In‐law			13		
	Friend			5		
Ego‐alter contact frequency^a^		1	6		3.96	1.53
Ego‐alter tie duration (years)		1	84		27.25	17.07

^a^ From 1 ‘Less often than several times a year’ to 6 ‘Everyday’.

Table 2 also presents the key characteristics of alters and ego‐alter ties included in the regression models and that are known to be associated with support provision. The mean age of alters is 44 years. About 27% of the alters live in the same household as ego. About 16% live in a different household located in the same municipality, 21% live in a different municipality that is less than 20 km away from where ego lives, 24% live in a municipality that is more than 20 km away and 12% live in another country. In terms of role relationship to the respondent, 21% of alters are categorised as children, 18% as extended family members (grandparents, aunts, uncles, cousins, siblings' partners, stepfamily members), 15% as siblings, 14% as parents, 13% as partners and 13% as in‐laws (parents‐in‐law, siblings‐in‐law and children‐in‐law). Friends considered as family members account for about 6% of all the important family members. We included in this category a few residual cases, such as co‐workers, neighbours, friends' family (e.g., a friend's spouse) or nannies/au pair that were too few to constitute a separate group. Contact frequency was operationalised with the question ‘*how often do you have contact to the named person, be it in person, by phone or by e‐mail, etc.’*, with the categories (6) every day, (5) almost every day, (4) several times a week, (3) several times a month, (2) several times a year and (1) less often. Mean contact frequency is 3.95. The tie duration was measured as a continuous variable. The mean duration is 27 years.

## 5 Results

Table 3 presents the results of the multilevel binary logistic regression models, in which *n*_*a*_ = 3345 ego‐alter ties (Level 1) are clustered within *n*_*e*_ = 549 egocentric networks (Level 2). The models predict whether an ego‐alter dyad involves reciprocal exchange of support as opposed to being non‐supportive or non‐reciprocal. They are run separately for material and emotional support. Statistical environment R and the lme4 package was used for analysis (R Core Team 2016). Positive coefficients indicate higher probabilities for the provision of reciprocal support compared to the reference categories; negative coefficients indicate lower probabilities. Models 0 are the empty models with intercept only. Models 1 estimate the effects of alter and tie characteristics (alter‐level covariates). Models 2 add the ego‐level characteristics and Models 3 add the network‐level characteristics. We discuss the results of all models together for material and emotional support. Lower values of AIC, BIC and −2 log likelihood statistics indicate a better model fit as the number of covariates increases from Model 0 to Model 3.

**TABLE 3:** Multilevel analysis of reciprocity in material and emotional support.

	Material support				Emotional support			
	Model 0	Model 1	Model 2	Model 3	Model 0	Model 1	Model 2	Model 3
(Intercept)	1.55^***^ (0.10)	0.89 (0.50)	−0.28 (0.79)	−2.31^**^ (0.85)	0.99^***^ (0.08)	−1.96^***^ (0.48)	−2.55^***^ (0.77)	−4.62^***^ (0.82)
Alter gender (ref: Male)		−0.02 (0.12)	−0.04 (0.12)	−0.06 (0.12)		0.95^***^ (0.11)	0.95^***^ (0.11)	0.93^***^ (0.11)
Alter age (ref: 31–50 years)								
0–17 years		−3.88^***^ (0.31)	−3.73^***^ (0.31)	−3.69^***^ (0.30)		−1.28^***^ (0.26)	−1.14^***^ (0.26)	−1.10^***^ (0.26)
18–30 years		−0.49^*^ (0.22)	−0.48^*^ (0.22)	−0.46^*^ (0.22)		−0.23 (0.20)	−0.22 (0.20)	−0.24 (0.20)
51–65 years		0.36 (0.20)	0.32 (0.20)	0.36 (0.20)		−0.09 (0.18)	−0.13 (0.18)	−0.09 (0.18)
66–100 years		−0.40 (0.23)	−0.50^*^ (0.23)	−0.47^*^ (0.23)		−0.83^***^ (0.22)	−0.93^***^ (0.22)	−0.91^***^ (0.22)
Ego‐alter residential distance (ref: Same municipality)								
Live in the same household		−1.95^***^ (0.33)	−2.04^***^ (0.32)	−2.07^***^ (0.33)		−1.30^***^ (0.28)	−1.34^***^ (0.28)	−1.27^***^ (0.28)
Another municipality < 20 km		−0.07 (0.24)	−0.09 (0.24)	−0.15 (0.23)		0.02 (0.21)	0.04 (0.21)	−0.04 (0.21)
Another municipality > 20 km		−0.35 (0.24)	−0.36 (0.24)	−0.38 (0.24)		0.16 (0.21)	0.17 (0.21)	0.06 (0.21)
Other country		−0.67* (0.29)	−0.73* (0.29)	−0.93^**^ (0.30)		0.24 (0.26)	0.15 (0.27)	0.03 (0.27)
Alter's role relationship with ego (ref: Child)								
Partner		0.04 (0.32)	0.10 (0.32)	0.23 (0.32)		1.66^***^ (0.30)	1.72^***^ (0.30)	1.81^***^ (0.30)
Father/mother		−0.71^*^ (0.32)	−1.03^**^ (0.32)	−1.04^**^ (0.32)		−0.22 (0.26)	−0.44 (0.27)	−0.46 (0.27)
Brother/sister		−1.40^***^ (0.27)	−1.71^***^ (0.27)	−1.70^***^ (0.27)		−0.90^***^ (0.23)	−1.13^***^ (0.24)	−1.14^***^ (0.24)
Extended family		−1.34^***^ (0.26)	−1.29^***^ (0.26)	−1.27^***^ (0.26)		−1.23^***^ (0.24)	−1.23^***^ (0.24)	−1.18^***^ (0.24)
In‐law		−1.26^***^ (0.30)	−1.10^***^ (0.30)	−1.07^***^ (0.30)		−1.31^***^ (0.28)	−1.20^***^ (0.28)	−1.22^***^ (0.28)
Friend		−1.85^***^ (0.34)	−1.77^***^ (0.34)	−1.49^***^ (0.33)		0.70^*^ (0.33)	0.74^*^ (0.33)	0.93^**^ (0.32)
Ego‐alter contact frequency		0.77^***^ (0.09)	0.78^***^ (0.09)	0.73^***^ (0.09)		0.83^***^ (0.08)	0.83^***^ (0.08)	0.75^***^ (0.08)
Ego‐alter tie duration (years)		0.01 (0.01)	0.03^***^ (0.01)	0.03^***^ (0.01)		0.02^*^ (0.01)	0.03^***^ (0.01)	0.03^***^ (0.01)
Ego gender (ref: Male)			−0.26 (0.25)	0.03 (0.23)			0.13 (0.24)	0.46^*^ (0.21)
Ego age (ref: 31–50 years)								
18–30 years			0.36 (0.38)	0.44 (0.34)			0.45 (0.36)	0.41 (0.31)
51–65 years			−0.26 (0.31)	0.04 (0.28)			−0.24 (0.30)	−0.09 (0.26)
66–100 years			−1.79^***^ (0.37)	−1.19^***^ (0.34)			−1.07^**^ (0.36)	−0.69^*^ (0.32)
Ego social class (ref: Upper‐grade service class)								
Unskilled workers			−1.05^*^ (0.49)	−1.02^*^ (0.44)			−1.04^*^ (0.47)	−1.04^*^ (0.41)
Skilled workers			−0.35 (0.34)	−0.42 (0.31)			−0.22 (0.33)	−0.34 (0.29)
Small business owners			−0.88^*^ (0.44)	−0.57 (0.40)			−0.26 (0.43)	0.03 (0.37)
Lower‐grade service class			0.03 (0.36)	−0.24 (0.32)			0.04 (0.34)	−0.39 (0.30)
Ego health status			0.42^**^ (0.14)	0.31^*^ (0.13)			0.20 (0.13)	0.13 (0.12)
Ego past migration			−0.07 (0.26)	0.01 (0.25)			0.17 (0.26)	0.32 (0.24)
Ego degree of urbanisation (ref: Cities)								
Towns and suburbs			−0.21 (0.29)	−0.26 (0.26)			−0.16 (0.29)	−0.26 (0.25)
Rural areas			0.65 (0.34)	0.54 (0.31)			−0.28 (0.32)	−0.59^*^ (0.28)
Network size (number of family members including ego) (ref: Small)								
Medium				0.74^*^ (0.29)				0.13 (0.27)
Large				0.65^*^ (0.31)				0.63^*^ (0.29)
Network density (ratio of existing to possible ties) (ref: Low)								
Medium				1.53^***^ (0.25)				1.78^***^ (0.24)
High				3.81^***^ (0.35)				3.54^***^ (0.31)
Network spatial dispersion (ref: All members in the same household)								
Average distance: < 5 km				0.02 (0.50)				0.02 (0.47)
Average distance: 5–20 km				0.34 (0.48)				0.86 (0.46)
Average distance: 20–100 km				0.28 (0.46)				1.28^**^ (0.44)
Average distance: 100+ km				0.69 (0.46)				0.66 (0.44)
AIC	3468.29	2767.04	2725.42	2598.41	3908.14	3202.53	3201.53	3055.49
BIC	3480.52	2883.23	2914.99	2836.90	3920.37	3318.72	3391.11	3293.99
Log likelihood	−1732.15	−1364.52	−1331.71	−1260.20	−1952.07	−1582.27	−1569.77	−1488.75
*n*_*a*_	3345	3345	3345	3345	3345	3345	3345	3345
*n*_*e*_	549	549	549	549	549	549	549	549
Var: Idnet (intercept)	2.68	5.81	4.87	3.35	2.29	5.23	4.87	3.16

*** *p* < 0.001.

** *p* < 0.01.

* *p* < 0.05.

Models 0 are a significant improvement for both material and emotional support compared with the fit of a single alter‐level null binary logistic model (Log‐likelihood ratio test: *p* < 0.001, not shown). In other words, the chance of reciprocally exchanging material and emotional support significantly varies from ego to ego and a multilevel approach is worthwhile to account for the clustering of alters within egocentric networks. The intra‐class correlation coefficients (ICCs) of 0.45 and 0.41 respectively for material and emotional support confirm a high degree of clustering.

The results of Models 1 show that respondents are more likely to reciprocally exchange material and emotional support with family members they are in frequent contact with (in person and remotely). In spatial terms, reciprocity in material support decreases with residential distance for non‐household family members, whereas reciprocity in emotional support remains unaffected. Reciprocity in both types of support is also less likely with household members compared to family members living nearby (within the same municipality). The results change when contact frequency is excluded from the models (not shown). The decrease in reciprocity with distance becomes stronger and is observed for both material and emotional support. The negative effect of living in the same household on material support is no longer significant, while for emotional support, the effect becomes significant and positive, indicating higher chances of mutual support when family members live in the same household compared to those living in the same municipality.

These results deserve consideration. In line with prior research (Plickert et al. 2007), we observe a decay in reciprocity as distance increases, but only for material support when we control for the amount of contact. This kind of support is more dependent on physical presence and where sizeable travel effort may deter providing help. The tendency for lower reciprocity in emotional support with far‐flung family members is explained by the lower amount of contact with them. The household penalty observed when controlling for contact may be explained by the fact that in‐household ties are qualitatively different from ties to family members living elsewhere. When it comes to assistance with money, childcare, housework or everyday problems, participants may not perceive household members as ‘support’ providers but rather as part of the routine organisation of household chores. In this view, support is more likely to be associated with help coming from outside the household.

Another result is that respondents are more likely to reciprocally exchange material and emotional support with family members aged 31–50 than with younger and older ones. Compared to ties with a child, ties with a parent, sibling, extended family member, in‐law and friend are more likely to involve reciprocal exchanges of material support. Emotional support is more likely to be reciprocally exchanged with the partner and friends, and less likely with siblings, extended family members and in‐laws. Mutually exchanging emotional support is also more likely with family members the respondents have known for many years. Finally, like for one‐way emotional support, gender is important for reciprocity. Reciprocal exchange of emotional support is more likely with female than with male family members. We did not find such gender difference for material support.

The results of Models 2 show that few characteristics of the respondents (ego) have significant effects on the likelihood that they reciprocally exchange support with their family members. In spatial terms, respondents' past migration and degree of urbanisation have no significant effect. People who moved away from their region of origin were as likely as those who stayed to report exchanging reciprocal support with their family members when controlling for other factors. We observe, however, that participants older than 65 years are less likely to reciprocally exchange support with family members than younger participants. Sensitivity analysis (see Appendix Table A2) shows that older participants are more likely than younger ones to declare that they would give emotional and material support, but would not receive it back. Compared to people in the higher‐grade service class, unskilled workers are less likely to reciprocally exchange material and emotional support. Small business owners are less likely to mutually exchange material support. People are also more likely to reciprocally exchange material support as their health quality is higher, probably because exchanging help with goods and services requires good health. In sum, people with higher socio‐economic and health status are more likely to engage in mutual exchange of support, arguably because they have more resources to share. When including individual characteristics in the models, the effect of tie duration becomes significant for reciprocity in material support.

Models 3 include the effects of network size, density and spatial dispersion on the likelihood of the ties to be mutually supportive. The larger the network, the more likely individuals are to reciprocally exchange material and emotional support within each tie. Citing more family members increases the chances for reciprocal exchanges, as previously found by Plickert et al. (2007). Ego‐alter ties are also more likely to be reciprocally supportive in tightly‐knit networks where alters support, influence and annoy one another than in sparsely‐connected family networks. Finally, the spatial dispersion of family networks is important for reciprocal exchange of emotional support. Compared to individuals with all their family members living in the same household, individuals are more likely to reciprocally exchange emotional support in spatially dispersed family networks with an average residential distance between family members of 20–100 km. Although not statistically significant, there is a similar tendency for smaller (less than 20 km) and larger (100+ km) spatial dispersion. When including network characteristics in the models, two ego‐level effects become significant for reciprocity in emotional support. Respondents in city centres are more likely than rural residents to have family members available for reciprocal emotional support, and so do female participants compared to male participants.

## 6 Discussion

This study examined how space shapes and is shaped by direct reciprocity of material and emotional support in families using multilevel modelling. Considering space as inherent characteristics of individuals, ties and families, we argue that mutual support between two family members is not only about how close or far away they live from one another, and how they stay in contact despite physical separation, but also about the characteristics of the entire family, including its spatial dispersion. Using a national sample of adults in Switzerland who listed important family members and available support, we have found that a substantial proportion of reported ties involve non‐reciprocal exchanges. In more than a quarter of ties, material support is not reciprocated, and in over a third, emotional support is not reciprocated. As discussed previously for the parent‐child bond (Hollstein 2005), important family relationships can be non‐reciprocal, or even non‐supportive. How is this variation related to spatial factors?

With respect to our first research question, we found that individuals' past migration and degree of urbanisation have weak effects on support reciprocity. Migrants are as likely as non‐migrants to report having family members available for reciprocal support. An urban‐rural difference emerges only for reciprocity in emotional support when controlling for network characteristics, with city residents more likely than rural respondents to report family members available for reciprocal emotional support. This pattern suggests that urban advantages in mobility resources may facilitate access to emotional—but not material—support, while norms governing material aid or institutional alternatives operate similarly across residential settings.

The answer to our second research question is that reciprocal exchange of support varies with spatial proximity to family members, but not quite as expected. Consistent with evidence on one‐way support provision (Hagge and Schacht 2024; Mok and Wellman 2007), reciprocity in family relationships declines with residential distance—but only in the case of material support. When controlling for contact quantity, mutual exchange of emotional support does not vary with distance for non‐household members. Moreover, reciprocity in material and emotional support is less likely with household members compared to family members living nearby. While mutual exchange of material support appears to depend more heavily on physical proximity, our findings suggest that reciprocity in emotional support can be sustained over distance through telecommunications and occasional visits. This aligns with research on spatial mobility (e.g., Holdsworth 2013; Urry 2012) and transnational families (Baldassar 2007; Halatcheva‐Trapp et al. 2019). Another possible explanation is a selection effect: strong and emotionally close relationships may be more likely to be named as important family members when living far from home. Conversely, co‐resident family members may be included due to shared living arrangements, regardless of whether support is mutually exchanged. A further interpretation of the observed household penalty effect—when controlling for contact—is that respondents may not perceive household members as sources of support, but rather as participants in the routine division of household responsibilities. In this view, support is more readily associated with help received from outside the household.

With respect to our third research question, multilevel analysis shows that sustaining mutually supportive ties is not only about distance and contact with individual family members. It is also about the spatial dispersion of the whole family. Contrary to the idea that having a family dispersed across different places may hinder support exchange, we find that individuals are more likely to reciprocally exchange emotional support with family members when embedded in spatially dispersed families compared to those in families where people live together. In line with the literature on transnational and diasporic families (Baldassar 2007; Baldassar and Merla 2013), these geographically dispersed families are capable of maintaining solidarity and organise care and mutual support across long distances. Another interpretation, from a Maussian perspective, is that reciprocal support itself sustains family relationships over distance. Reciprocating support may serve as a way of actively ‘doing family’—a dynamic that becomes even more important when family members are physically separated.

Besides spatial effects, we observe that reciprocal support is more likely to occur in family relationships when these are embedded in cohesive family networks with high density, as predicted by the theoretical approaches to reciprocity (Adloff and Mau 2006; Hollstein 2005; Mauss 2001[1925]; Offer 2012). This finding is particularly relevant to the study of the interplay between space and social networks. Some may argue that spatial dispersion matters because it hinders dense connections between group members. We observe that the effect of spatial dispersion cannot be subsumed or totally explained by an effect of density of connections, and that both network and spatial contexts should be considered.

Our findings show that space cannot be reduced to dyadic distances between family members considered in isolation but understood in the broader family and network contexts. The finding that reciprocal emotional support is more likely in family ties within spatially dispersed networks than within local ones provides new empirical evidence that some individuals and families build support systems across borders and large distances (Baldassar and Merla 2013; Lubbers et al. 2020). Our findings also support previous evidence on one‐way support exchange that geographical distance is not necessarily detrimental to support provision that does not require regular physical contact, such as emotional or financial aid (Herz 2015; Madhavan et al. 2017; Mok and Wellman 2007; Viry 2012).

Certain aspects of this research must be considered critically. First, we regarded Switzerland as a case to study. However, because it is a small, affluent and well‐connected country, Switzerland is only partly comparable with other national contexts. It remains to be investigated whether reciprocity in family exchanges operates the same way in other geographical and cultural contexts. Second, we could not quantify the amount, quality and perceived fairness of support exchanged, nor distinguish financial help, minor and major services with the measure of support available. Similarly, cultural differences in family support were not investigated. Third, our focus was on direct (one‐to‐one) exchange of support between respondents and their family members, and not on more complex forms of generalised reciprocity. Further research could measure family network dispersion at finer‐grained scales. However, alternative measures to mean distance, like median distance, confirmed that the results discussed are robust and not merely driven by the choice of spatial metric. Future work could also analyse reciprocal exchange in larger structures than dyads (e.g. triads). From a structural position, reciprocation between A and B may be affected by whether A and B have reciprocal relationships with a third person C. In this study, we accounted for these dependencies by including structural dimensions of networks. The recent methodological development of exponential random graph models (ERGMs) with egocentric network data may offer new directions for future research (Krivitsky and Morris 2017; Vega Yon et al. 2021). Finally, reciprocity should not be seen as static, but as process. We need more longitudinal data, both quantitative and qualitative, to better understand how reciprocity in family relationships changes over time and as people move away or come closer. The data used only provided information on support and distance between family members at a certain point in time but did not capture the sequence of events and the causal chain between them, with no information on the timing of moves or the reasons for moving (e.g., family needs or conflict). We considered space to be an inherent characteristic of relationships, individuals and families, with the statistical models treating relationships as nested within individuals and personal networks. Other processes by which space and reciprocity are co‐evolving over time are possible, for instance through the influence of some places (e.g., family home). Longitudinal data will enhance our ability to understand the two‐way interactions between space and reciprocity, for example when people who fail to reciprocate may move away to escape the ‘burden’ of reciprocity (Offer 2012).

## Conflicts of Interest

The authors declare no conflicts of interest.

## Data Availability

The data that support the findings of this study are openly available in SWISSUBase at https://www.swissubase.ch/en/.

## Appendix

**TABLE A1:** Logit models of participating in the follow‐up survey (Model 1), of participating in the follow‐up survey and answering the questions on material/emotional support (Model 2), of participating in the follow‐up survey and answering all the questions related to the variables included in the final models (Model 3).

	Model 1	Model 2	Model 3
(Intercept)	0.16 (0.33)	0.19 (0.33)	0.05 (0.33)
Gender (ref: Male)	0.58*** (0.13)	0.49*** (0.13)	0.55*** (0.13)
Age (ref: 18–30 years)			
31–50 years	0.22 (0.19)	−0.02 (0.19)	−0.11 (0.19)
51–65 years	0.86*** (0.21)	0.47* (0.20)	0.21 (0.20)
66–100 years	0.69*** (0.23)	0.04 (0.22)	−0.23 (0.23)
Education (ref: ISCED 0–3 short)			
ISCED 3	0.19 (0.18)	0.52** (0.18)	0.59** (0.18)
ISCED 4 + 5B (tertiary vocational)	0.08 (0.23)	0.31 (0.22)	0.42 (0.23)
ISCED 5A + 6 (tertiary general)	0.40 (0.24)	0.76** (0.24)	1.03*** (0.24)
Social class (ref: Upper‐grade service class)			
Unskilled workers	−0.34 (0.27)	−0.65* (0.27)	−0.65* (0.27)
Skilled workers	−0.11 (0.22)	−0.33 (0.22)	−0.37 (0.22)
Small business owners	−0.45 (0.25)	−0.41 (0.25)	−0.40 (0.25)
Lower‐grade service class	−0.28 (0.21)	−0.31 (0.21)	−0.38 (0.21)
Swiss citizenship (ref: Yes)			
No	−0.76*** (0.17)	−0.78*** (0.17)	−0.76*** (0.18)
Residential linguistic region (ref: French‐speaking)			
German‐speaking	−0.40* (0.16)	−0.56*** (0.16)	−0.63*** (0.15)
Italian‐speaking	0.15 (0.41)	−0.26 (0.39)	−0.06 (0.39)
Partnership status (ref: Stable partner)			
No stable partner	−0.05 (0.15)	−0.12 (0.15)	−0.19 (0.15)
Number of children	0.01 (0.05)	0.00 (0.05)	0.04 (0.05)
AIC	1494.04	1533.27	1518.44
BIC	1579.89	1619.12	1604.29
Log likelihood	−730.02	−749.63	−742.22
Deviance	1460.04	1499.27	1484.44
*n*_*e*_	1153	1153	1153

^***^ *p* < 0.001.

^**^ *p* < 0.01.

^*^ *p* < 0.05.

**TABLE A2:** Multilevel analysis of reciprocal exchange of any form of support (material or emotional) (left) and one‐way support with ego giving one or both forms of support but not receiving any (right).

	Reciprocal exchange of material or emotional support				One‐way exchange with ego giving material or emotional support and not receiving any support			
	Model 0	Model 1	Model 2	Model 3	Model 0	Model 1	Model 2	Model 3
(Intercept)	2.38^***^ (0.13)	1.22^*^ (0.58)	0.08 (0.89)	−2.38^*^ (0.94)	−2.70^***^ (0.13)	−2.31^***^ (0.60)	−2.01^*^ (0.85)	−0.83 (0.93)
Alter gender (ref: Male)		0.34^*^ (0.14)	0.34^*^ (0.14)	0.30^*^ (0.14)		−0.19 (0.14)	−0.18 (0.15)	−0.15 (0.14)
Alter age (ref: 31–50 years)								
0–17 years		−3.09^***^ (0.32)	−2.93^***^ (0.32)	−2.85^***^ (0.32)		3.28^***^ (0.33)	3.08^***^ (0.33)	2.98^***^ (0.33)
18–30 years		−0.59^*^ (0.26)	−0.57^*^ (0.26)	−0.55^*^ (0.26)		0.87^**^ (0.27)	0.82^**^ (0.27)	0.78^**^ (0.27)
51–65 years		0.46^*^ (0.23)	0.41 (0.23)	0.45^*^ (0.23)		−0.45 (0.27)	−0.36 (0.27)	−0.40 (0.26)
66–100 years		−0.31 (0.26)	−0.44 (0.27)	−0.37 (0.26)		0.53 (0.28)	0.70^*^ (0.29)	0.64^*^ (0.28)
Ego‐alter residential distance (ref: Same municipality)								
Live in the same household		−2.66^***^ (0.39)	−2.76^***^ (0.39)	−2.71^***^ (0.39)		1.83^***^ (0.39)	1.92^***^ (0.39)	2.02^***^ (0.39)
Another municipality < 20 km		−0.30 (0.27)	−0.31 (0.27)	−0.42 (0.27)		0.22 (0.28)	0.20 (0.28)	0.30 (0.28)
Another municipality > 20 km		−0.24 (0.27)	−0.23 (0.27)	−0.30 (0.27)		0.29 (0.28)	0.22 (0.28)	0.30 (0.28)
Other country		−0.52 (0.33)	−0.58 (0.34)	−0.85^*^ (0.34)		0.55 (0.33)	0.59 (0.34)	0.79^*^ (0.35)
Alter's role relationship with ego (ref: Child)								
Partner		0.60 (0.42)	0.72 (0.42)	0.84^*^ (0.41)		−0.91 (0.52)	−1.10^*^ (0.52)	−1.21^*^ (0.52)
Father/mother		−1.34^***^ (0.36)	−1.64^***^ (0.37)	−1.63^***^ (0.36)		0.55 (0.40)	0.82^*^ (0.41)	0.86^*^ (0.40)
Brother/sister		−1.62^***^ (0.31)	−1.93^***^ (0.32)	−1.90^***^ (0.31)		1.19^***^ (0.32)	1.48^***^ (0.33)	1.49^***^ (0.32)
Extended family		−1.72^***^ (0.30)	−1.63^***^ (0.30)	−1.59^***^ (0.29)		1.18^***^ (0.30)	1.03^***^ (0.31)	0.98^**^ (0.30)
In‐law		−1.78^***^ (0.35)	−1.57^***^ (0.35)	−1.54^***^ (0.34)		1.38^***^ (0.36)	1.09^**^ (0.36)	1.07^**^ (0.36)
Friend		−1.26^**^ (0.41)	−1.16^**^ (0.41)	−0.86^*^ (0.40)		0.86^*^ (0.43)	0.73 (0.43)	0.49 (0.43)
Ego‐alter contact frequency		1.01^***^ (0.11)	1.02^***^ (0.11)	0.93^***^ (0.10)		−0.76^***^ (0.11)	−0.76^***^ (0.11)	−0.70^***^ (0.10)
Ego‐alter tie duration (years)		0.01 (0.01)	0.03^**^ (0.01)	0.03^**^ (0.01)		−0.01 (0.01)	−0.02^*^ (0.01)	−0.02^*^ (0.01)
Ego gender (ref: Male)			−0.26 (0.28)	0.07 (0.25)			0.32 (0.25)	0.06 (0.23)
Ego age (ref: 31–50 years)								
18–30 years			0.46 (0.42)	0.48 (0.37)			−0.30 (0.38)	−0.31 (0.36)
51–65 years			−0.23 (0.35)	0.03 (0.31)			0.36 (0.31)	0.24 (0.29)
66–100 years			−1.76^***^ (0.42)	−1.14^**^ (0.37)			1.57^***^ (0.37)	1.28^***^ (0.35)
Ego social class (ref: Upper‐grade service class)								
Unskilled workers			−1.16^*^ (0.55)	−1.08^*^ (0.48)			0.51 (0.48)	0.60 (0.45)
Skilled workers			−0.27 (0.39)	−0.33 (0.34)			0.09 (0.34)	0.21 (0.32)
Small business owners			−0.71 (0.49)	−0.29 (0.43)			0.30 (0.43)	0.04 (0.40)
Lower‐grade service class			−0.12 (0.40)	−0.47 (0.35)			−0.28 (0.35)	−0.01 (0.33)
Ego health status			0.41^**^ (0.16)	0.29^*^ (0.14)			−0.11 (0.14)	−0.07 (0.13)
Ego past migration			−0.18 (0.30)	−0.05 (0.27)			0.01 (0.26)	−0.15 (0.25)
Ego degree of urbanisation (ref: Cities)								
Towns and suburbs			−0.21 (0.33)	−0.24 (0.29)			0.16 (0.29)	0.20 (0.27)
Rural areas			0.52 (0.38)	0.32 (0.33)			−0.13 (0.33)	−0.05 (0.31)
Network size (number of family members including ego) (ref: Small)								
Medium				0.69^*^ (0.32)				0.05 (0.32)
Large				0.83^*^ (0.34)				0.25 (0.34)
Network density (ratio of existing to possible ties) (ref: Low)								
Medium				1.90^***^ (0.28)				−1.06^***^ (0.25)
High				4.24^***^ (0.41)				−2.90^***^ (0.38)
Network spatial dispersion (ref: All members in the same household)								
Average distance: < 5 km				0.11 (0.56)				0.27 (0.55)
Average distance: 5–20 km				0.77 (0.55)				−0.73 (0.54)
Average distance: 20–100 km				0.70 (0.52)				−0.57 (0.52)
Average distance: 100+ km				0.96 (0.52)				−0.54 (0.52)
AIC	2778.73	2240.48	2212.56	2085.47	2347.00	1899.09	1891.88	1827.35
BIC	2790.96	2356.67	2402.14	2323.96	2359.23	2015.28	2081.45	2065.84
Log likelihood	−1387.37	−1101.24	−1075.28	−1003.73	−1171.50	−930.55	−914.94	−874.67
*n*_*a*_	3345	3345	3345	3345	3345	3345	3345	3345
*n*_*e*_	549	549	549	549	549	549	549	549
Var: Idnet (intercept)	2.98	6.49	5.59	3.34	2.00	3.81	3.39	2.36

^***^ *p* < 0.001.

^**^ *p* < 0.01.

^*^ *p* < 0.05.
